# Potential of [^11^C]UCB-J as a PET tracer for islets of Langerhans

**DOI:** 10.1038/s41598-021-04188-6

**Published:** 2021-12-28

**Authors:** Emmi Puuvuori, Johanna Rokka, Per-Ola Carlsson, Zhanchun Li, Jonas Eriksson, Olof Eriksson

**Affiliations:** 1 Science for Life Laboratory, Department of Medicinal Chemistry, Uppsala University, Dag Hammarskjöldsv 14C, 3rd floor, 75183 Uppsala, Sweden; 2 Department of Public Health and Caring Sciences, Uppsala University, Uppsala, Sweden; 3 Department of Medical Cell Biology, Uppsala University, Uppsala, Sweden

**Keywords:** Target identification, Target validation, Diagnostics, Endocrine system and metabolic diseases, Diagnostic markers

## Abstract

Biomarkers for the measurement of islets of Langerhans could help elucidate the etiology of diabetes. Synaptic vesicle glycoprotein 2 A (SV2A) is a potential marker reported to be localized in the endocrine pancreas. [^11^C]UCB-J is a novel positron emission tomography (PET) radiotracer that binds to SV2A and was previously evaluated as a synaptic marker in the central nervous system. Here, we evaluated whether [^11^C]UCB-J could be utilized as a PET tracer for the islets of Langerhans in the pancreas by targeting SV2A. The mRNA transcription of SV2A was evaluated in human isolated islets of Langerhans and exocrine tissue. In vitro autoradiography was performed on pancreas and brain sections from rats and pigs, and consecutive sections were immunostained for insulin. Sprague–Dawley rats were examined with PET-MRI and ex vivo autoradiography at baseline and with administration of levetiracetam (LEV). Similarly, pigs were examined with dynamic PET-CT over the pancreas and brain after administration of [^11^C]UCB-J at baseline and after pretreatment with LEV. In vivo radioligand binding was assessed using a one-compartment tissue model. The mRNA expression of SV2A was nearly 7 times higher in endocrine tissue than in exocrine tissue (*p* < 0.01). In vitro autoradiography displayed focal binding of [^11^C]UCB-J in the pancreas of rats and pigs, but the binding pattern did not overlap with the insulin-positive areas or with ex vivo autoradiography. In rats, pancreas binding was higher than that in negative control tissues but could not be blocked by LEV. In pigs, the pancreas and brain exhibited accumulation of [^11^C]UCB-J above the negative control tissue spleen. While brain binding could be blocked by pretreatment with LEV, a similar effect was not observed in the pancreas. Transcription data indicate SV2A to be a valid target for imaging islets of Langerhans, but [^11^C]UCB-J does not appear to have sufficient sensitivity for this application.

## Introduction

The current understanding of beta cell mass (BCM) within the Islets of Langerhans of the pancreas in each individual during the progression of human diabetes is still unclear. Contemporary understanding of the progression of human diabetes is based on extrapolation from animal models or histology of human tissue biopsies. Repeatable and noninvasive measurement of the islet mass would open opportunities to improve our knowledge of the etiology of diabetes and enable the assessment of treatments designed to protect or restore beta cell mass or function.

An auspicious option would be to use medical imaging techniques. For example, hybrid positron emission tomography (PET) combined with computed tomography (CT) has sufficient sensitivity to potentially visualize pancreatic islets. Before the scan, a patient is injected with a radiolabeled compound, often a protein or small molecule that will bind to a specific receptor. Afterwards, the patient is placed in a PET scanner, where the photons generated from positron emission and radionuclide decay will be detected in the organ of interest. An image of physiological uptake of the tracer is reconstructed, which can then be fused together with the anatomical CT image for more precise localization.

There are several promising beta cell PET tracers under evaluation, but currently, there are no clinically validated biomarkers available to image BCM^1^. A major challenge is defining beta cell surface markers, which would not be present on other closely related pancreatic neuroendocrine cells, such as alpha cells.

One possibility is to harness already established PET tracers that are selective for the endocrine pancreas. This would rapidly enable imaging and quantification of the islet mass, which could serve as a substitute for BCM. Previously, our group successfully imaged islet masses with [^11^C]5-hydroxytryptophan, a precursor of serotonin present in all endocrine cells in the pancreas, but the demanding radiochemistry process makes widespread use of the tracer challenging^2–4^. A recently developed nanobody targeting dipeptidyl peptidase 6 (DPP6) has shown successful results in rodent imaging and in vitro^5^. A possible drawback of nanobodies is the unclear nature of their immunogenicity due to their camelid derivate origin, which might hinder the process of the clinical use of the tracer^6^.

Since beta cells and neurologic tissues have common cellular receptors and transporters, neurological imaging tracers could potentially be utilized to image endocrine islets^7^. Synaptic vesicle glycoprotein 2 (SV2) is a transmembrane protein present in neurons and on secretory vesicles in endocrine cells^8^. The SV2 family consists of three protein isoforms, SV2A, -B and -C, which are expressed in separate locations in insulin-secreting cells^8^. SV2A and SV2C are larger in size than SV2B (742, 727 and 683 amino acids) and share more similarities considering their amino acid chains^9^. Despite the resemblances in structures, all three isoforms possess specific expression patterns. In the human, mouse and pig brain, SV2A is found in all areas, followed by SV2B, with a few exceptions (parts of the thalamus and substantia nigra)^10^. SV2C, however, has most of the expression in the brain limited to the basal ganglia^11^.

SV2A is also reported to have strong expression in human and rodent pancreatic islets but not the exocrine pancreas, presumably since the neuroendocrine pancreas shares many phenotypic traits with neuronal cells^8,12^. Therefore, PET imaging and quantification of SV2A density in the pancreas could potentially serve as a proxy of islet mass.

In recent years, several radiotracers have been developed to target SV2A based on the antiepileptic agent levetiracetam (LEV)^13^. Some of the most promising compounds, such as [^11^C]UCB-A, ^14^[^18^F]UCB-H and [^11^C]UCB-J, were derived from compound screening performed by UCB Pharma (Braine-l'Alleud, Belgium)^15^. In comparison with [^11^C]UCB-A and [^18^F]UCB-H, [^11^C]UCB-J has been the most extensively used and displays optimal pharmacokinetics and properties for quantification^15,16^. [^11^C]UCB-J has high affinity for human SV2A (8.15 pKi, ≈7 nM), moderate affinity for SV2C (7 pKi, ≈ 100 nM) and low affinity for SV2B (5.7 pKi, ≈ 2 µM), and^17^ [^11^C]UCB-J is available for both preclinical (affinity for rat SV2A is 25 nM) and clinical studies and is widely used as a synaptic marker for the central nervous system (CNS)^16,17^.

In a previous communication, we described a proteomic screening of the Human Protein Atlas database^18^, with the aim of finding novel islet- and beta cell-specific proteins. SV2A was one of the initial hits during the investigation, even though it was islet, rather than beta cell specific. Furthermore, [^11^C]UCB-J was recently evaluated in an effort to identify novel islet markers in a retrospective screening of neuroendocrine tracers and their binding in the pancreas^7^. [^11^C]UCB-J was found to exhibit visible pancreatic uptake of SUV≈1.6 in nondiabetic human individuals. This was higher than abdominal reference tissue spleen. Additionally, SUV 1.6 can be considered a reasonable magnitude of uptake for a tracer specifically targeting the pancreatic islets, which consist of only 2–3% of the cells in the tissue.

In this study, our hypothesis was that if the SV2A protein is selective for the islet of Langerhans, then [^11^C]UCB-J could be utilized as a PET tracer for visualization of the endocrine pancreas.

## Materials and methods

### Transcriptomics

The RNA transcriptomics was performed on isolated human islet and exocrine tissue from five donor pancreases stored at − 70 °C, as well as fresh frozen pancreatic tissue embedded in OCT compound (Sakura Finetek, Alphen aan den Rijn, The Netherlands). The use of human tissues from Uppsala Biobank (registration #827) was approved by the Regional Ethics Board, Uppsala, Sweden (now Swedish Ethical Review Authority) (2011/473, Ups 02–577, 2015/401) and were anonymized, collected, and treated according to local institutional and Swedish national rules and regulations. The need for informed consent was renounced by the Regional Ethics Board in Uppsala.

RNA was extracted using the RNeasy mini kit (Qiagen) according to the manufacturer’s instructions. Disruption was conducted using a 3 mm steel grinding ball (VWR, Radnor, PA) and vortexing. Concentration and RNA integrity (RIN) were determined by Qubit 2.0 Fluorometer (Life Technologies) and Agilent 2100 Bioanalyzer (Agilent Technologies, Santa Clara, CA), respectively. The purity of the samples was confirmed by an A260/A280 value over 2.0 using a Nanodrop (Thermo Scientific, Wilmington, DE). Samples with RIN values above 7.5 were sequenced by Illumina HiSeq2000 and 2500 (Illumina, San Diego, CA) using the standard Illumina RNA-seq protocol with a read length of 2 × 100 bases. The details of the analysis of mRNA levels in this dataset have been described previously ^19^.

### Radiosynthesis of [^11^C]UCB-J

[^11^C]UCB-J was synthesized using commercially available precursor compound (Pharmasynth AS) and a one-step synthesis method in THF/water and formulated in 10% ethanol in phosphate saline solution (PBS) as reported previously^20^. With this method, the radioactivity yield of [^11^C]UCB-J was 1500 ± 600 MBq, molar activity 340 ± 190 MBq/nmol and radiochemical purity > 99.9% at the end of the synthesis (n = 15).

### In vitro autoradiography binding studies

Adjacent frozen Sects. (10 µm) of rat and pig pancreas as well as rat brain and excised INS1 xenografts (insulinoma/beta cell model) were prepared on cryostat (Cryostar NX70, Thermo fisher scientific) at − 20 °C and mounted on adhesion glass slides (Superfrost Plus, Menzel-Gläser, Germany) to be stored at − 20 °C. For in vitro autoradiography (ARG), the frozen slides were first preincubated in phosphate buffered saline (PBS 150 ml, pH 7.4) at room temperature for 10 min and washed with MQ water for 1 min. Next, [^11^C]UCB-J in PBS and ethanol (1 MBq/ml) was added, and the sections were incubated for 40 min. To remove the excess buffer and unbound [^11^C]UCB-J, the slides were washed three times in PBS and once in MQ water and dried in a laboratory drying oven (Termaks, Bergen, Norway) for 10 min. A set of calibration standards were created for quantification by pipetting 10 μl drops of the same stock onto absorbent chromatography paper. The slides were then exposed to a freshly erased storage phosphor screen (BAS-MS, Fujifilm) for two half-lives of ^11^C (40 min) and then scanned on a phosphor imager (Amersham Typhoon FLA 9500 Phospor Imager, GE) with 4000 sensitivity and 25 µm pixel size. The images were analyzed in ImageJ (National Institutes of Health, US) software.

### Immunostaining for insulin

Adjacent sections of frozen in vitro autoradiography were immunostained for insulin expression. Sections were fixed in acetone (4 °C) for 5 min, allowed to dry for 15 min, and then washed in PBS and Dako washing buffer. Donkey serum (3% in PBS) was added for blocking and incubated for 20 min at RT, followed by overnight incubation with guinea pig anti-insulin 1:400 (Fitzgerald, Acton, MA, USA). Cat No. 20-IP30) at 4 °C. The next day, the sections were first washed in PBS 2 × 5 min and 1 × Dako washing buffer. Alexa 488-conjugated donkey anti-guinea pig secondary antibody (1:300, Jackson ImmunoResearch Laboratories, West Grove, PA, USA). Code No. 706–545-148) were added to the sections for 50 min at RT. Afterwards, the sections were rinsed 3 × 5 min PBS and then incubated with DAPI for 10 min to achieve a background. Finally, the sections were washed 2 × PBS and mounted in fluorescence mounting medium on protective glass.

### Animal handling

The procedures involving animal experimentation were approved by the Animal Ethics Committee of the Swedish Animal Welfare Agency and carried out in accordance with the ARRIVE and institutional guidelines (“Uppsala university guidelines on animal experimentation”, UFV 2007/724).

### PET-MRI imaging of rats

The tracer distribution was examined in Sprague–Dawley (SPRD) rats. The rats were anesthetized as described above and placed on a heated pad under the camera immediately after the [^11^C]UCB-J injections (22 MBq to 74 MBq). A 60 min dynamic nanoPET-MRI scan (Mediso Medical Imaging Systems, Hungary) over the pancreas (1.5–2.0 mm spatial resolution^21^, 212 × 212 × 239 matrix) was acquired with short T1-weighted axial (TR/TE 497/9.45 ms) and coronal (TR/TE 481/9.45 ms) sequences (0.4 mm spatial resolution, 63 slices) prior to a 30 min scan over the brain (TR/TE 300/9.45 ms, 38 slices), either at baseline (n = 4) or following preblocking (n = 3) with 30 mg/kg LEV in NaCl 0,9% (10 mg/ml) 30 min before the start of the scan. The rats were euthanized by CO_2_ after the scans. The PET images were reconstructed for attenuation and scatter correction using Tera-Tomo™ 3D (OSEM, Monte Carlo DOI estimation, correction for attenuation, scatter, randoms and dead time to even positron range: 4 iterations, 6 subsets, voxel size 0.4 mm^3^,0.3 mm^3^ resolution) reconstruction.

### Ex vivo autoradiography

Ex vivo autoradiography was performed on rats (n = 8) injected with [^11^C]UCB-J. Briefly, rats were anaesthetized with isoflurane (5% initially and afterwards 3% to maintain anesthesia), and [^11^C]UCB-J was administered by a single injection (46 MBq to 184 MBq, corresponding to a similar mass of the tracer) via the tail vein. The rats were sacrificed by CO_2_ 20 (n = 5), 40 (n = 1), 60 (n = 1) or 80 (n = 1) min after injection. Two of the rats euthanized 20 min post injection were pretreated with the blocking agent LEV (30 mg/kg) prior to radioactive compound.

Immediately after euthanasia, the brain, pancreas and spleen were excised, embedded in OCT media and snap frozen. Coronal slices of the pancreas and spleen and axial slices of 20 μm-thick brain sections were cut using a cryostat (Cryostar NX70, Thermo Fisher Scientific). The sections were mounted on Superfrost Plus microscope slides (Menzel-Gläser, Germany) and exposed similarly as described above against a phosphor (BAS-MS) imaging plate for 40 min at room temperature.

### PET-CT imaging of pigs

Healthy male and female locally bred pigs (n = 3, 26 ± 3 kg) were placed supine on a table and anaesthetized with fentanyl 5–10 mg/kg IV and maintained with a continuous IV infusion of ketamine 30 mg/kg/h, midazolam 0.1–0.4 mg/kg/h, and fentanyl 4 mg/kg/h. The pig’s vitals (blood glucose, oxygen saturation, ECG, arterial blood pressure, body temperature and end-tidal carbon dioxide (ETCO_2_)) were monitored throughout the whole study, and fluid homeostasis was maintained with a continuous infusion of Ringer acetate (10 ml/kg/h) and NaCl 0.9% (Fresenius Kabi AB, Sweden). An arterial catheter was inserted in the right carotid artery for blood sampling and blood pressure measurement, and an introducer was placed on a central venous catheter for administration of [^11^C]UCB-J and contrast media.

First, an attenuation CT scan (100 kV, 80–400 mA, noise index 10, rotation 0.5’’, full spiral, slice thickness 3.75 mm, pitch 0.98:1, recon diameter 50 mm) was acquired using a digital 4-ring system, 64-slice CT with a 198 mm axial field of view (FOV). A 90 min (4 mm spatial resolution, 33 frames: 12 × 10’’, 6 × 30’’, 5 × 2’, 5 × 5’, 5 × 10’) dynamic PET scan (Discovery MI, GE Healthcare) over the pancreas and a 30 min static scan over the brain were performed after bolus injection of [^11^C]UCB-J (260–350 MBq). The scans were repeated after IV pretreatment (1 h before tracer injection) of LEV 20 mg/kg in 100 ml of NaCl 0,9% as slow infusion (400 ml/h), and a new bolus of [^11^C]UCB-J was administered approximately 4 h after the first injection. Arterial blood samples were acquired during the dynamic scans to obtain the tracer volume in both the plasma and whole blood by measuring the activity on a gamma well counter. The pancreas was visualized independently by late arterial (15 s) and venous phase contrast-enhanced CT (Omnipaque 350 + 40 ml NaCl, 3.5 ml/s, bolus tracking on descending aorta 100 HU threshold) to assist segmentation of PET images. The PET images were reconstructed using an iterative VPFX-S algorithm (GE Healthcare) (OSEM, Time of Flight, Resolution recovery: 3 iterations, 16 subsets, 3 mm postfilter and 256 × 256 matrix).

### PET data analysis

Volumes of interest were defined manually over the organs on SUV corrected transaxial projections using PMOD software (PMOD Technologies LLC, Zürich, Switzerland). Regional time activity curves (TACs) were obtained segmenting the pancreas, brain and adrenal glands for target organs and the spleen and muscle as negative references. The graphs were outlined on Microsoft Excel (Microsoft Office) and GraphPad Prism (GraphPad Software Inc., La Jolla, CA, USA). All data are presented as the mean and standard deviation of the mean. Comparisons between baseline and blocking studies were assessed by Student's t-test (two-tailed), where *p* < 0.05 was considered significant.

## Results

### SV2A isoform is localized in the islets of the pancreas

**Figure 1:**
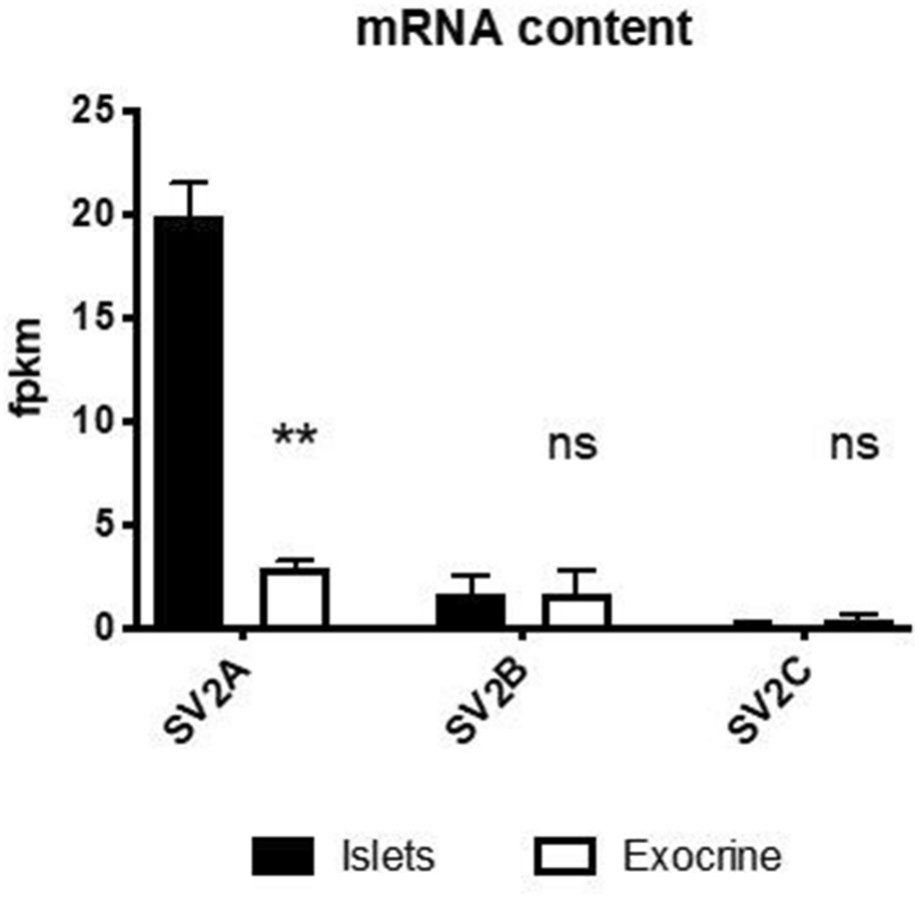
Transcription levels of SV2 isoforms in the pancreatic compartments. SV2A transcription was higher (fragments per kilobase million, fpkm) in isolated human islets of Langerhans than in the exocrine pancreas. The transcription of isoforms SV2B and SV2C was lower than that of SV2A, and the levels in islets and exocrine pancreas did not differ.

SV2A mRNA transcription was almost sevenfold higher in isolated human islets of Langerhans (n = 5) than in exocrine tissue (n = 3) from the same subjects (Fig. 1). Isoform SV2B and SV2C transcription did not differ between islets and exocrine tissue and was generally low, especially for SV2C.

### In vitro autoradiography of [^11^C]UCB-J and insulin staining did not demonstrate corresponding patterns

**Figure 2:**
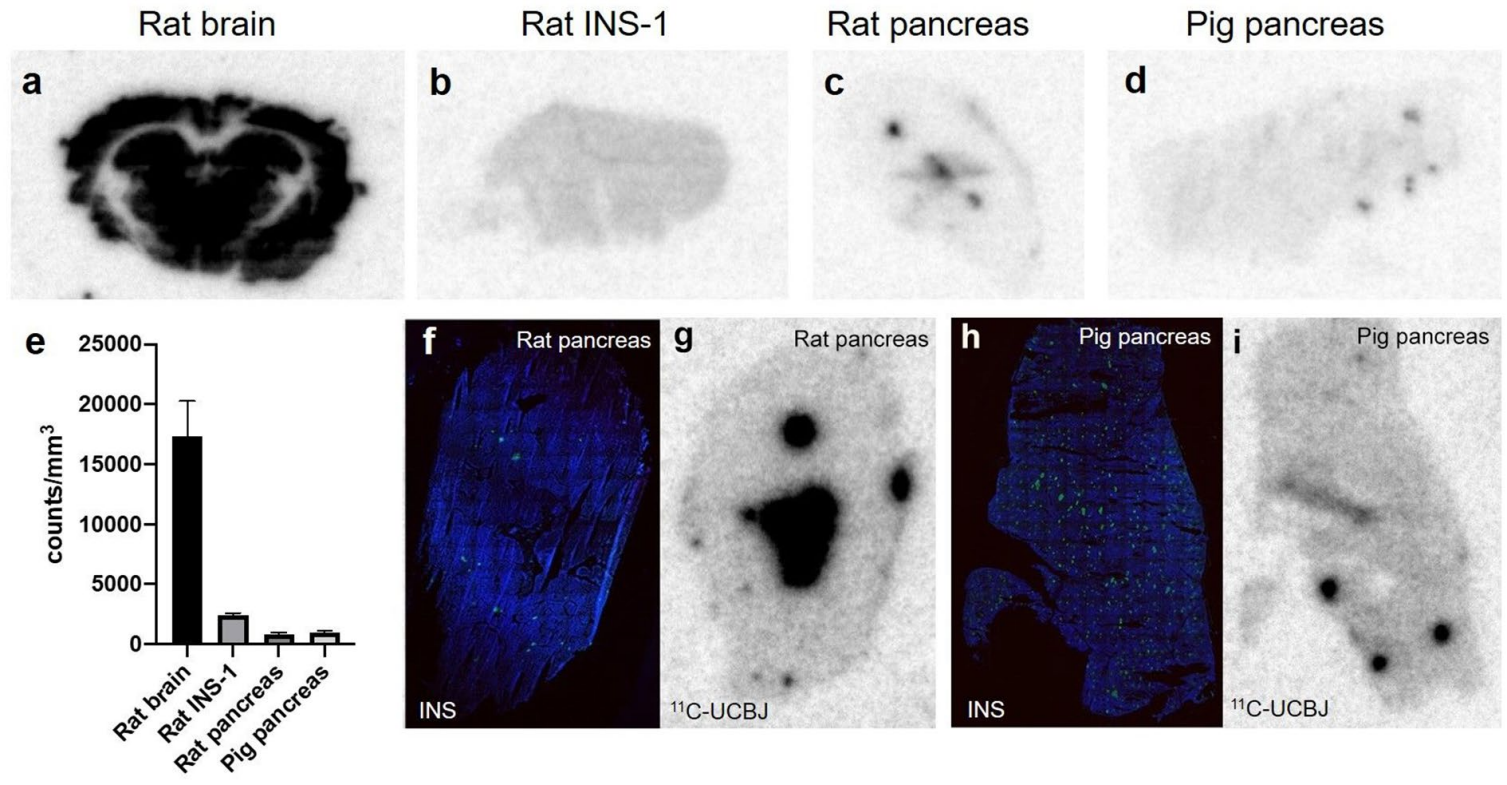
In vitro autoradiographic binding studies in the brain and pancreas. Representative autoradiograms of [^11^C]UCB-J binding in sections of rat brain (**a**), rat insulinoma (INS-1) tumor (**b**), rat pancreas (**c**) and pig pancreas (**d**). The binding intensities were comparable, as all representative sections were from the same autoradiographic plate. Quantified binding was higher in the brain and INS-1 than in the rat pancreas (**e**). Rat (**g**) and pig (**i**) pancreases demonstrated visually discrete focal binding patterns that were suspected to represent islets, but correlative staining (blue color for DAPI, green color for insulin) for insulin (**f**, **h**) indicates poor overlap between [^11^C]UCB-J binding and islets of Langerhans.

In vitro binding of [^11^C]UCB-J was high in rat brain sections, a positive control tissue with known expression of SV2A (Fig. 2a and e). Binding in the pancreas from rats (Fig. 2c) and pigs (Fig. 2d) exhibited a low exocrine background and focal binding pattern, suggestive of binding to Langerhans islets. In line with this reasoning, binding in INS-1 sections (Fig. 2b) (i.e., a model of pure insulinoma/beta cells) was generally higher than the exocrine background but in a similar range as the focal uptake seen in rat pancreas.

However, immunostaining of consecutive pancreas sections for insulin (i.e., marking insulin-positive islets of Langerhans, color green) showed no clear overlap between the focal uptake autoradiography patterns (Fig. 2g and i) and insulin-positive regions in either rats (Fig. 2f) or pigs (Fig. 2h).

### [^11^C]UCB-J binding in rat pancreas and brain

**Figure 3:**
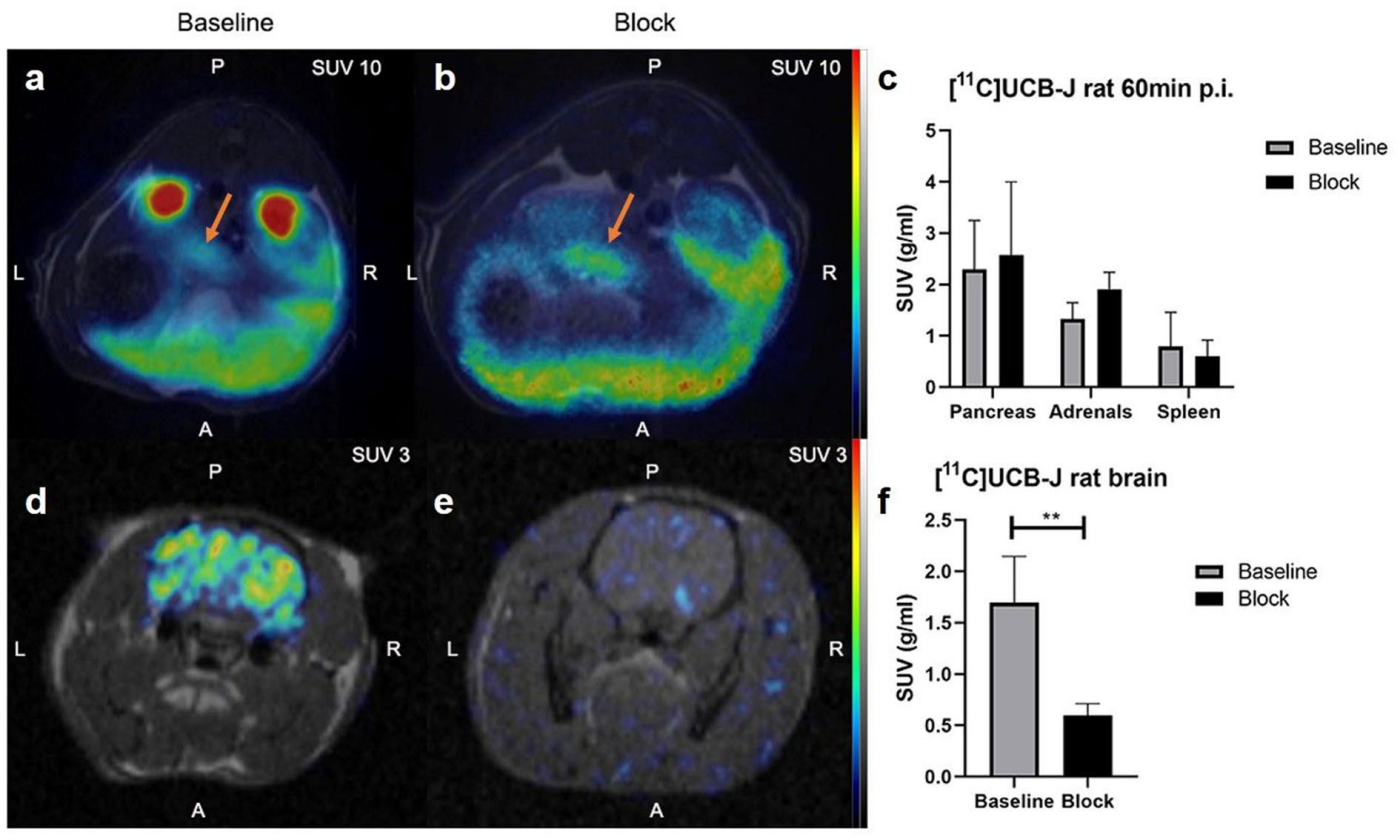
In vivo PET-MRI imaging in rat. Representative abdominal (**a**) 60 min p.i. and brain (**d**) PET-MRI images at baseline. Pretreatment did not abolish binding in pancreas (orange single arrow) (**b**), while blocking was efficient in the brain (**e**), as demonstrated by representative images. Quantification of tissue uptake verified poor or negligible blockade of abdominal binding with 30 mg/kg LEV (**c**), while binding in the brain was reproducibly inhibited (**f**).

In vivo PET-MRI imaging demonstrated binding of [^11^C]UCB-J in abdominal neuroendocrine tissues pancreas and adrenals (Fig. 3a and c), higher than, reference tissue spleen which is reported to have no expression of SV2A (See full kinetic data in Supplementary Figs. S1 and S2). Similarly, the positive control tissue brain exhibited strong binding of [^11^C]UCB-J (Fig. 3d and f). Pretreatment with LEV abolished binding in the brain (*p* < 0.01, Fig. 3e and f) but not in the pancreas or adrenal glands (Fig. 3b and c).

**Figure 4:**
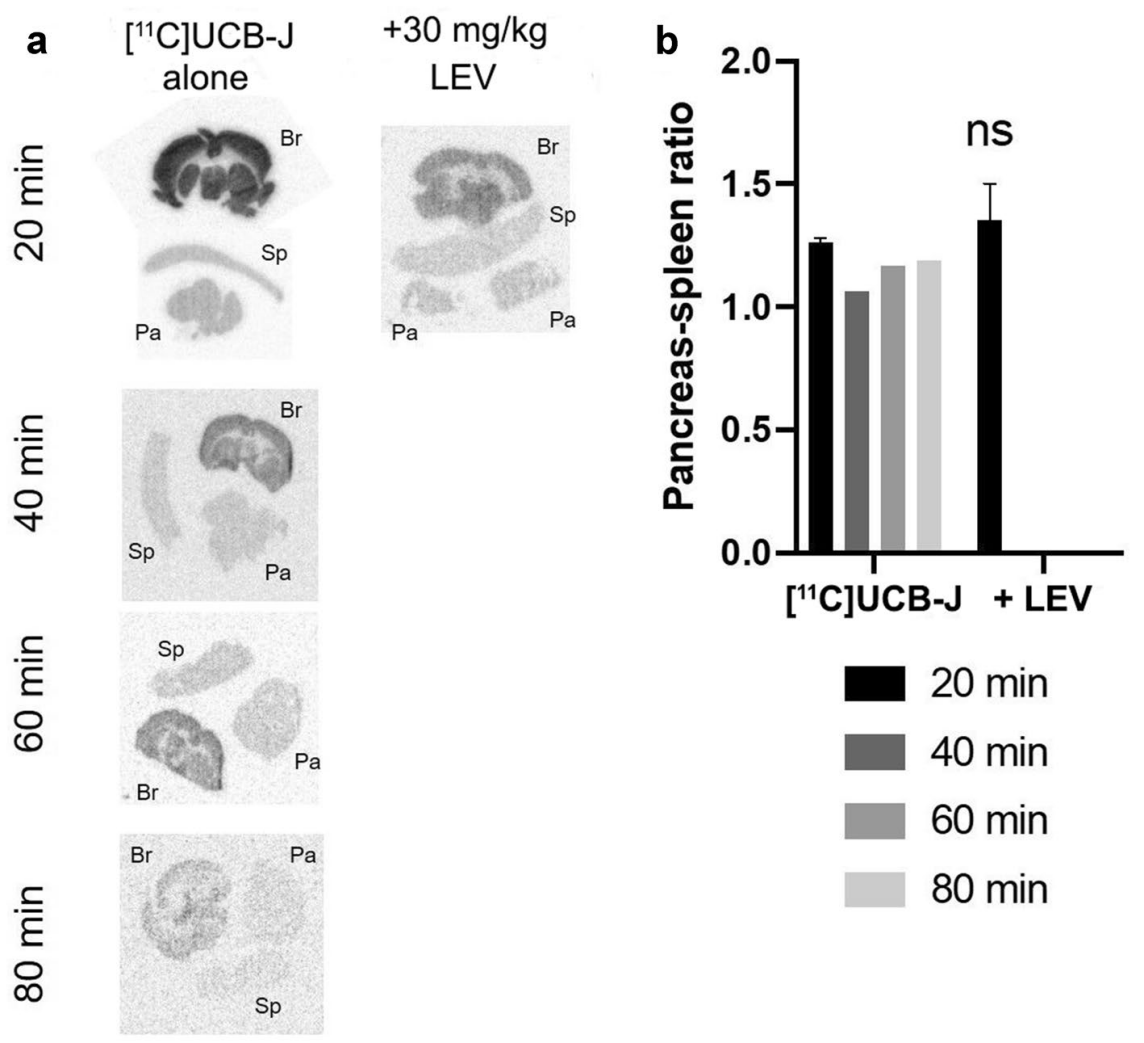
Ex vivo autoradiography in rat tissues. Postmortem autoradiography clearly demonstrated strong brain binding of [^11^C]UCB-J across all time points (**a**), which could be abolished by pretreatment with 30 mg/kg LEV. The binding in the pancreas was low and did not indicate islet-specific targeting. Pancreas-spleen ratio was barely higher (and not blockable) than negative control tissue spleen (**b**).

The in vivo binding was confirmed by ex vivo autoradiography of tissue sections postmortem. The brain demonstrated strong binding compared with the reference tissue spleen. Similar to in vitro autoradiography, the binding was inhibited by pretreatment with LEV in the brain (Fig. 4a, b). There was no internal structure of [^11^C]UCB-J binding present in the pancreas, as would be expected of specific targeting of the islets compared with exocrine pancreas (Fig. 4a). The pancreas to spleen uptake ratio was above 1.0, but uptake in the pancreas was not blockable by pretreatment with LEV, as was also observed for the in vivo PET data.

### [^11^C]UCB-J binding in pig pancreas and brain

**Figure 5:**
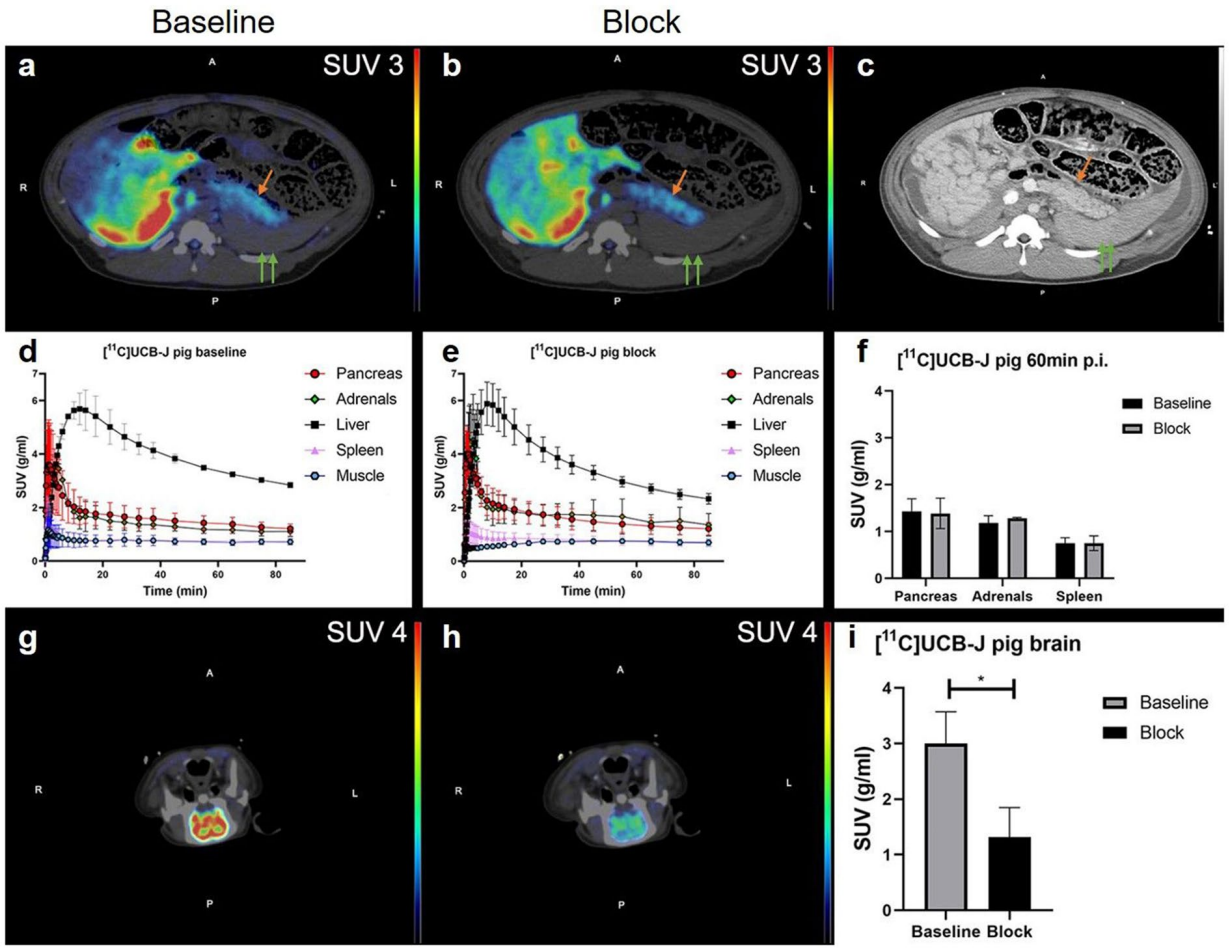
In vivo PET-CT imaging in pigs. Baseline (**a**) and post LEV treatment (**b**) SUV-corrected 55–90 min averaged representative images of the abdomen in pigs. The pancreas, spleen (green double arrows) and adrenal glands were reliably identified and segmented by support from contrast-enhanced CT images (**c**). Corresponding TACs of mean SUV values at baseline (**d**) and after block (**e**) did not show any decrease in pancreas (**f**) binding of [^11^C]-UCBJ at 60 min p.i. However, the positive control tissue brain demonstrated strong binding at baseline (**g**), which was decreased in the same animal after administration of 20 mg/kg LEV (**h**) (representative, comparable images shown). Blocking of the brain binding of [^11^C]UCB-J was observed in all three examined pigs (**i**).

The pig pancreas (Fig. 5a, b) was identified and delineated with assistance from contrast-enhanced CT images (Fig. 5c). After administration to healthy pigs, [^11^C]UCB-J was rapidly distributed from blood to organs, and uptake (Fig. 5a and d) in the pancreas (SUV = 1.43 ± 0.27) and adrenal neuroendocrine tissues (SUV = 1.18 ± 0.15) was noticeable. The uptake in the reference tissue spleen (SUV = 0.75 ± 0.12) remained low at all times. High accumulation in excretion tissue liver (SUV = 4.18 ± 1.22) reached the peak at 14 min p.i. and started gradually clearing afterwards. Pretreatment with LEV did not decrease the binding in the pancreas (SUV = 1.39 ± 0.32) or adrenal glands (SUV = 1.67 ± 0.66) compared with the baseline scan (Fig. 5b, e and f). After pretreatment with LEV, the activity concentration in the liver peaked earlier and that in the adrenal glands was higher than that in the baseline scan (Fig. 5e). The positive reference region brain exhibited strong binding at baseline (SUV = 3.00 ± 0.57) (Fig. 5g), which was decreased by LEV pretreatment (SUV = 1.32 ± 0.53, *p* < 0.05) (Fig. 5h–i).

## Discussion

A fully functional islet-specific tracer would provide important information considering changes in the endocrine pancreas in several diseases, most notable diabetes. PET tracers targeting specifically the pancreatic beta cells are under intense investigation as this technology could yield direct non-invasive assessment of the health and number of insulin-producing cells. However, as this endeavor has proven challenging^1^, we believe it is similarly important to evaluate existing possibilities for targeting the entire population of endocrine cells in the pancreas (e.g. the islets of Langerhans with all cell subtypes).

In this study, we investigated the potential of the neurotracer [^11^C]UCB-J for visualization of pancreatic islets by PET imaging. The study was motivated by the strong SV2A immunoreactivity in the pancreatic islets, as established in the literature, in combination with the emergence of clinically available PET tracers for SV2A.

First, the transcriptomic analysis of the SV2 isoforms verified that SV2A was present in the islets and absent from the exocrine pancreas, in line with earlier reports on SV2A protein expression in the pancreas. Furthermore, SV2B and especially SV2C transcription was very low or absent in the islets, which is counter to some reports that have described SV2C protein localization and function in human beta cells^8^. The role of SV2A (and the other isoforms) in neuroendocrine cells is still not completely clarified but is believed to be associated with docking of secretory vesicles to the cell membrane in preparation for exocytosis of neurotransmitters or hormones such as insulin.

The transcriptomic data reinforced earlier immunohistochemistry data that indicated that SV2A could be a potential target for imaging islets of Langerhans, given a suitable PET tracer with sufficient specificity for SV2A. Therefore, we next evaluated the pancreatic binding of [^11^C]UCB-J, which targets human SV2A with high affinity (≈7 nM), in rats and pigs.

Initial data in rat in vitro autoradiography appeared promising, given the focal uptake pattern in the pancreas as well as the observation of generally higher binding in pure beta cells (INS-1 xenografts) than in the exocrine pancreatic background. However, the tracer binding pattern did not correlate with insulin staining (e.g., presence of functional islets) and in vitro autoradiography. Furthermore, in vivo binding in the pancreas could not be blocked by LEV. In contrast, uptake in the positive control tissue brain was clearly visible, and its binding could be completely abolished by LEV pretreatment both in vivo and ex vivo. The affinity of [^11^C]UCB-J for rat SV2A (≈ 25 nM) is worse than that for human SV2A (≈ 7 nM). This severely hampers the sensitivity of [^11^C]UCB-J for detecting dispersed and diluted islets in the pancreas, particularly in rats. Therefore, the tracer was also evaluated in pigs, which is a more translational model in metabolic disease.

In pigs, a similar focal binding pattern was observed in the in vitro autoradiography pancreatic sections, with the same magnitude as in rats. Again, immunostaining for insulin did not show a convincing overlap of the autoradiography signal. Finally, in vivo PET-CT scanning in pigs demonstrated clear visualization of the pancreas, as well as other abdominal neuroendocrine tissue adrenal glands. As expected from several previous studies^13,17^, brain uptake (SUV = 3.00 ± 0.5) was relatively omnipresent in the pig brain. Brain binding of [^11^C]UCB-J was blockable by LEV, indicating high affinity to porcine SV2A (the K_D_ in this species has not been reported previously). Furthermore, pancreatic uptake after 1 h (SUV≈1.5–2) was in the range previously reported in the human pancreas^7^. However, as in rats, neither pancreatic nor adrenal binding could be decreased by LEV pretreatment. This was clearly not due to insufficient exposure to LEV, as almost all binding sites in the brain (behind the BBB no less) could be inhibited in the same animals.

The lack of blockable binding in the pancreas in pigs is difficult to reconcile with the sum of observations but could be due to a few reasons. First, pancreatic uptake could simply be nonspecific, but this is counterintuitive to the much lower binding observed in the spleen, which has a similar blood supply and magnitude of perfusion. It seems peculiar that two similar tissues with similar tracer distributions should have such distinctly different nonspecific binding. There is a possibility that some of the tracer uptake in pancreas could be due to circulating radiometabolites (e.g. metabolite of UCB-J incorporating the Carbon-11 nuclide). The lack of measurement of [^11^C]UCB-J radio-metabolites in blood is a general drawback with this study, especially in the pig imaging data, which could otherwise potentially be analyzed by kinetic modeling using the metabolite corrected arterial blood plasma input function. Such an analysis could potentially shed light on the respective contribution of the in vivo pancreatic binding of [^11^C]UCB-J, with regards to specific receptor binding to SV2A, non-specific binding and radiometabolite accumulation. However, as also our in vitro and ex vivo autoradiography data indicates limited sensitivity of the currently evaluated technique specifically for pancreatic islet imaging, we do not expect radiometabolite accumulation to be a major confounding factor. The limited sensitivity of [^11^C]UCB-J in pancreas is further in line with the conclusion in the report by Bini et Al^7^. Second, LEV may exert a blocking effect in the CNS but not the pancreas. Again, this seems unlikely, since the situation normally is reversed (lower exposure in brain compared with the periphery due to the BBB). Finally, LEV only binds to SV2A, while [^11^C]UCB-J also exhibits some modest cross-reactivity to the SV2C isoform (≈100 nM)^17^. In theory, this could result in residual [^11^C]UCB-J binding in SV2C-positive tissues, also after LEV blockade. However, our transcription data indicate only negligible expression of SV2C in islets or exocrine pancreas. Nevertheless, SV2C protein localization in islets and beta cells has been reported, which could potentially yield some minor [^11^C]UCB-J binding.

We recently presented longitudinal data pancreatic uptake of the endocrine PET marker ^11^C-5-hydroxy-tryptophan from initial diagnosis of T1D^22^. Interestingly, we observed only a small decline in binding over the first two years after T1D diagnosis, indicating negligible loss of the total pancreatic endocrine mass over this period. Furthermore, ^11^C-5-hydroxy-tryptophan binding was decreased compared to non-diabetic individuals already at T1D onset. Clearly, further studies with islet specific PET markers are warranted to better understand these observations, but the main drawback with ^11^C-5-hydroxy-tryptophan is its complicated radiochemistry limiting its use to just a few centers worldwide. Here, we thus evaluated [^11^C]UCB-J as a potential clinically available alternative to ^11^C-5-hydroxy-tryptophan, as a marker for islets of Langerhans. Since Carbon-11 tracers require on-site cyclotron due to the short half-life, other potential options to target SV2A would be e.g. [^18^F] labeled UCB-J and SynVesT-1^23,24^.

An islet marker could potentially be used to study interventions or processes that affect either the number of alpha cells, beta cells or alter their ratio in the pancreas. This includes several recently proposed and established mechanisms in diabetes progression, e,g, dedifferentiation (decreasing beta cell mass but constant endocrine mass) and beta cell destruction (decreasing both beta cell and islet mass) respectively. Measuring the global mass of all endocrine cell types would also be crucial in a hypothetical opposite situation: if endocrine progenitor cells are induced to transdifferentiate into beta cells. Additionally, following up the survival of transplanted pancreatic islets safely in vivo without any alterations of the implanted cells would open a great opportunity to comprehend more about the mechanisms involved. In the islet transplantation setting an islet specific marker would also provide similar value as a beta cell specific PET marker, as post transplantation graft loss often affect the entire islets and not just the beta cell component.

A PET readout for total pancreatic endocrine cell mass should potentially be combined with other endpoints to give optimal information. For example, complementary assessments could be established estimations of beta cell function (e.g. oral or intravenous metabolic tests), beta cell death (e.g. methylated DNA). In a future perspective, one may also consider the synergistic value in combining several PET markers, e.g. for endocrine mass and beta cell mass, or endocrine mass and inflammation markers.

## Conclusion

In this study, we explored whether neurotracer [^11^C]UCB-J could be utilized as a PET tracer for the endocrine pancreas. We demonstrated SV2A mRNA transcription in islets but not in the exocrine pancreas. [^11^C]UCB-J revealed higher uptake in the pancreas than the surrounding tissues in rats and pigs, but binding could not be blocked with LEV. Furthermore, in vitro and ex vivo binding of [^11^C]UCB-J in the pancreas was not associated with insulin-positive areas in tissue. SV2A may still be a valid target for imaging islets of Langerhans, but [^11^C]UCB-J does not appear to have sufficient sensitivity for this application.

## Supplementary Information

Supplementary Figure 1.

Supplementary Figure 2.

Supplementary Legends.
