# Supplementary material for: Potential of [11C]UCB-J as a PET tracer for islets of Langerhans
Source: Sci Rep. 2021 Dec 28;11:24466. doi: 10.1038/s41598-021-04188-6 (PMC8714818; doi:10.1038/s41598-021-04188-6)
Supplement: Supplementary file 1 — Supplementary Figure 1. [file 41598_2021_4188_MOESM1_ESM.docx]

**Supplementary figure legends**

Supplementary figure S1. In vivo PET kinetics of rat during baseline. TAC of rat pancreas show increase in uptake during the first 10 min, of which starts to gradually decrease. The uptake in spleen and adrenals reach equilibrium around 30 min and remain lower compared with adrenal uptake throughout the whole study.

**Supplementary figure S2**. **In vivo PET kinetics of rat after blocking.** After blocking with LEV pancreas uptake follows the same pattern as during baseline, but slightly higher throughout. Adrenal uptake follows similar pattern as pancreas, with increased uptake after blocking. Spleen uptake remains on the same level as during baseline.
