# Supplementary figures and images for: Potential of [11C]UCB-J as a PET tracer for islets of Langerhans
Source: Sci Rep. 2021 Dec 28;11:24466. doi: 10.1038/s41598-021-04188-6 (PMC8714818; doi:10.1038/s41598-021-04188-6)

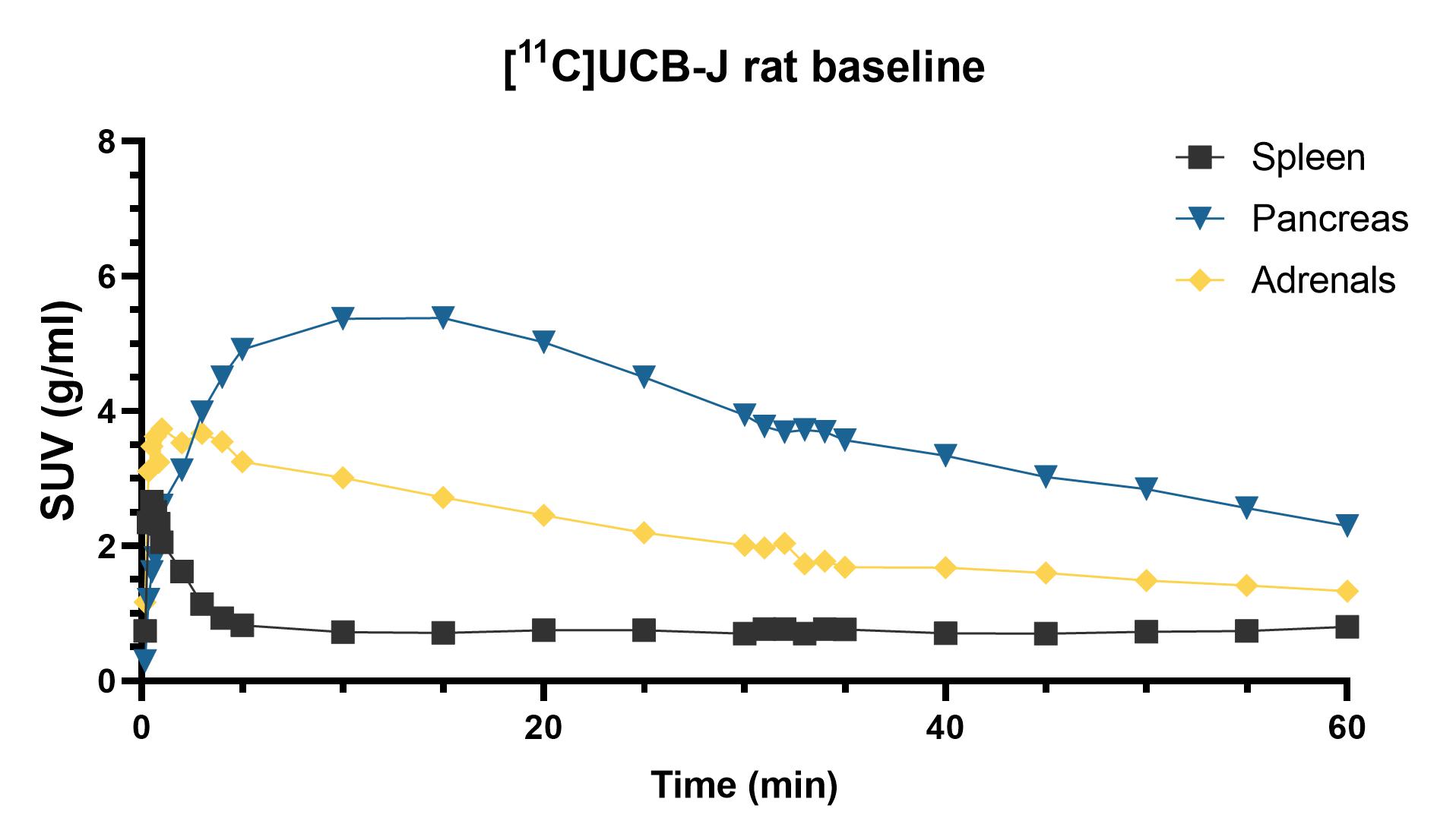

Supplement: Supplementary file 2 — Supplementary Figure 2. [file 41598_2021_4188_MOESM2_ESM.jpg]

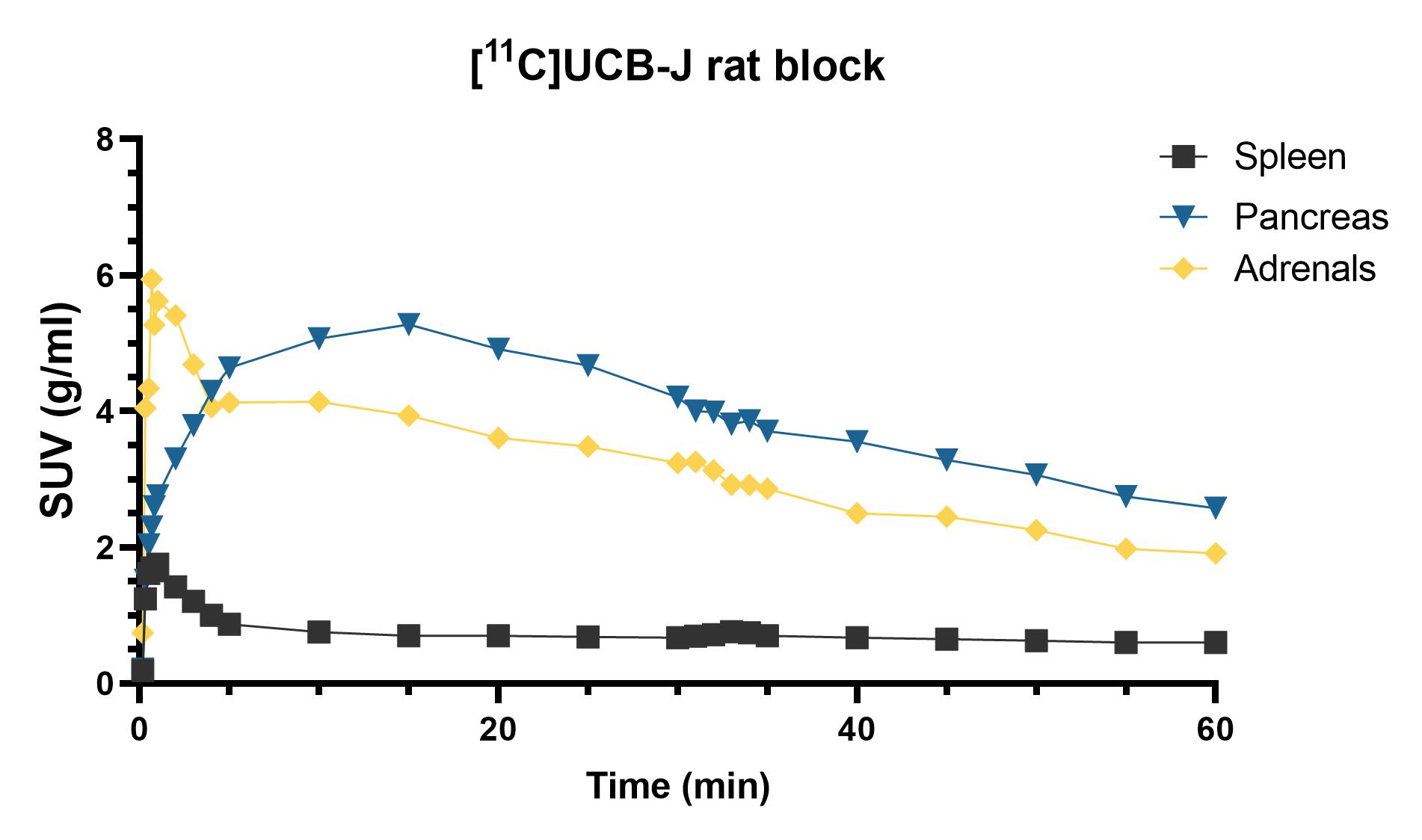

Supplement: Supplementary file 3 — Supplementary Legends. [file 41598_2021_4188_MOESM3_ESM.jpg]
